# Near-native, site-specific and purification-free protein labeling for quantitative protein interaction analysis by MicroScale Thermophoresis

**DOI:** 10.1038/s41598-018-23154-3

**Published:** 2018-03-21

**Authors:** Tanja Bartoschik, Stefanie Galinec, Christian Kleusch, Katarzyna Walkiewicz, Dennis Breitsprecher, Sebastian Weigert, Yves A. Muller, Changjiang You, Jacob Piehler, Thomas Vercruysse, Dirk Daelemans, Nuska Tschammer

**Affiliations:** 1 NanoTemper Technologies GmbH, Floessergasse 4, 81069 München, Germany; 2 Division of Biotechnology, Department of Biology, Friedrich-Alexander University Erlangen, Nuremberg Henkestr 91, 91052 Erlangen, Germany; 3 Division of Biophysics, Department of Biology, University Osnabrück, Barbarastr 11, 49076 Osnabrück, Germany; 4 KU Leuven Department of Immunology and Microbiology, Laboratory of Virology and Chemotherapy, Rega Institute for Medical Research, Herestraat 49, 3000 Leuven, Belgium

## Abstract

MicroScale Thermophoresis (MST) is a frequently used method for the quantitative characterization of intermolecular interactions with several advantages over other technologies. One of these is its capability to determine equilibrium constants in solution including complex biological matrices such as cell lysates. MST requires one binding partner to be fluorescent, which is typically achieved by labeling target proteins with a suitable fluorophore. Here, we present a near-native, site-specific *in situ* labeling strategy for MST experiments that enables reliable measurements in cell lysates and that has distinct advantages over routine covalent labeling techniques. To this end, we exploited the high-affinity interaction of tris-NTA with oligohistidine-tags, which are popular for purification, immobilization or detection of recombinant proteins. We used various DYE-tris-NTA conjugates to successfully label His-tagged proteins that were either purified or a component of cell lysate. The RED-tris-NTA was identified as the optimal dye conjugate with a high affinity towards oligohistidine-tags, a high fluorescence signal and an optimal signal-to-noise ratio in MST binding experiments. Owing to its emission in the red region of the spectrum, it also enables reliable measurements in complex biological matrices such as cell lysates allowing a more physiologically realistic assessment and eliminating the need for protein purification.

## Introduction

Robust and reliable determination of the affinity between a target molecule and its interaction partner is a critical step in many areas of biological, biochemical and biomedical research and technology. For example, early phases of drug discovery include steps such as target identification and validation, hit discovery and lead optimization. During all of these steps, quantitative characterization of intermolecular interaction affinity is highly necessary to develop novel and effective drugs for therapeutic interventions^1^. MicroScale Thermophoresis (MST) is a versatile method to quantify binding affinities in solution that is increasingly applied for interaction analysis^2,3^. In this technique a variation in the fluorescence signal is detected, which is a result of a temperature gradient induced by an infrared laser^4^. The extent of the variation in the fluorescence signal correlates with the binding of a ligand to the fluorescent target^5^; thus, MST can be used to quantify the interaction and to determine equilibrium dissociation constants (K_d_). One notable advantage of MST over other routinely used methods for the quantification of molecular binding events, such as SPR and ITC, is that it can also be used for the determination of K_d_ values in complex sample matrices like cell lysate and serum^4,6,7^. Although MST measurements can be performed using intrinsic fluorescence of proteins, labeling of the target proteins with a suitable fluorophore is required when using such complex samples. Unfortunately, in routine labeling techniques, the fluorophore is covalently attached to lysine residues using NHS- or to cysteine residues using maleimide chemistry. These labeling strategies are limited to purified proteins and cannot be applied in a mixture of several proteins or in complex biological matrices such as cell lysate or blood serum^8^. The generation of purified protein can be challenging, time-consuming and expensive, sometimes not even applicable for the protein of interest^9^. Moreover, it is not possible to predict where the fluorophore will bind to the protein. Hence, covalent labeling of a protein with NHS or maleimide conjugated dye can lead to inhomogeneous protein-dye conjugates, some of which might even display destabilization or loss of functionality^10^. Fortunately, in contrast, site-specific protein modification strategies allow structurally and stoichiometrically well-defined labeling with minimal perturbation of structural and functional integrity. Two things that have conquered modern life cell fluorescence imaging are the genetic fusion of fluorescent proteins and enzymes specifically engineered for posttranslational labeling^11^, but such relatively large tags are not always desired for quantitative interaction analysis. With the use of bioorthogonal conjugation reactions, labeling of non-purified proteins with high selectivity is possible, allowing rapid and cost-effective labeling^12^. Different site-specific labeling strategies have been proposed and applied, including co-translational introduction of unnatural or modified amino acids, or labeling via specific amino acid sequences, such as His-tag sequences and tetracysteine motifs^8,13–17^. Among these sequences, the His-tag is the most popular and widely used affinity tag for purification, immobilization or detection of proteins^18–21^.

**Figure 1:**
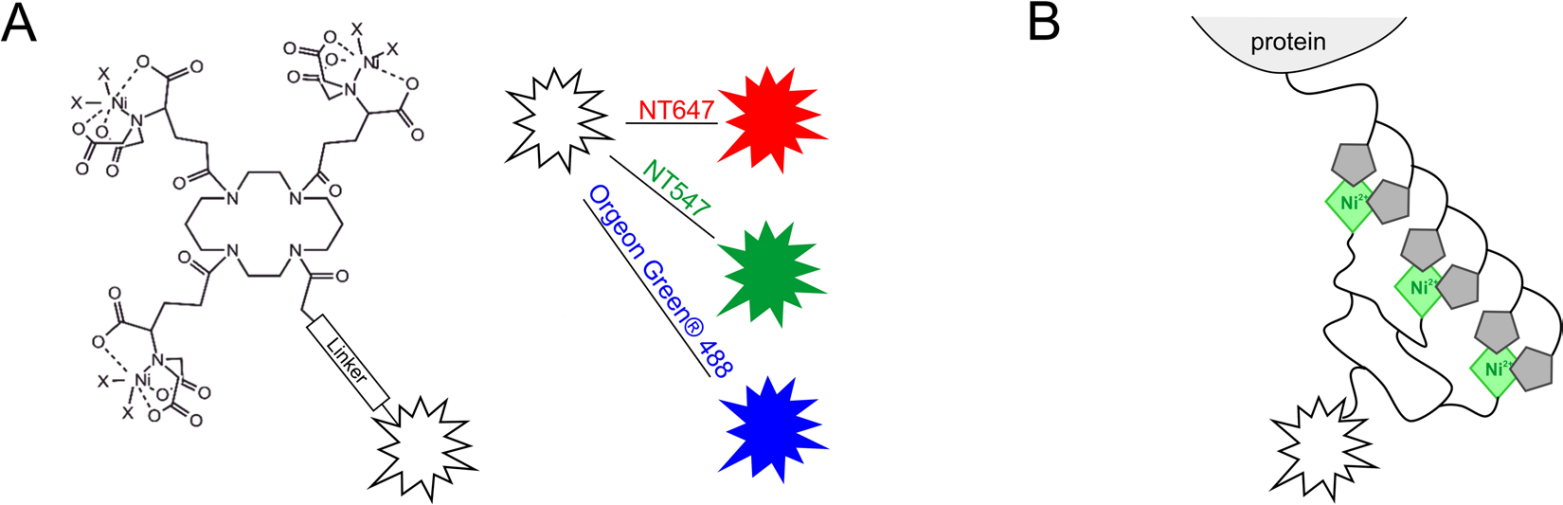
Labeling of His-tagged proteins via tris-NTA conjugates. (**A**) Chemical structure of the tris-NTA moiety conjugated to a fluorophore via a linker. Fluorophores are illustrated on the right: RED (NT647), GREEN (NT547) and BLUE (Oregon Green® 488). (**B**) Schematic representation of DYE-tris-NTA bound to a His-tagged protein. The conjugate is loaded with Ni(II) ions for the site-specific labeling of histidine-tagged proteins. Two remaining coordination sites of the NTA-complexed Ni(II) ions interact with histidine moieties of the protein’s His-tag.

The tris-NTA/His-tag system comprises one of the smallest high-affinity recognition elements known to date^22^. This interaction is based on the capacity of the histidine’s imidazole groups to form coordinative bonds with transition metal ions such as Ni(II). Chelators such as nitrilotriacetic acid (NTA)^23^ stably bind Ni(II) ions via three oxygen atoms and one nitrogen atom. The two remaining coordination sites of Ni(II) can each bind one histidine moiety of a His-tag (Fig. 1)^8^, yielding a molecular binding affinity of ~10 µM^24^. Tris-NTA is comprised of three NTA moieties coupled to a cyclic scaffold and thus can simultaneously bind six Histidine residues of a His_6_-tag, yielding subnanomolar binding affinity and a well-defined 1:1 stoichiometry^24^. Fast, stoichiometric binding of tris-NTA conjugates enabled *in situ* protein labeling of His-tagged proteins^25–28^ that was compatible with complex sample matrices including living cells^22,25,29–31^. These unique features make tris-NTA/oligohistidine interaction labeling an attractive candidate for quantitative protein interaction analysis by MST.

In this work, we present the application of tris-NTA-based labeling of His-tagged proteins for MST measurements. For this purpose, tris-NTA was conjugated to three different fluorescent dyes: RED (NT647), GREEN (NT547) and BLUE (Oregon Green® 488), providing fluorophores from different spectral regions (Fig. 1A). The dyes NT647 and NT547 are MST-optimized dyes, which are commercially available as NHS or maleimide derivatives for MST measurements. Our data highlights the versatility, robustness and superiority of the novel tris-NTA labeling approach for MST. Overall, the RED-tris-NTA conjugate (NT647-tris-NTA) arose as the optimal dye conjugate for this approach. This conjugate is characterized by a high affinity for His-tags, a high fluorescence signal and the best signal-to-noise ratio of all investigated DYE-tris-NTA conjugates. Owing to its red emission spectrum, it enables reliable measurements in complex biological matrices such as cell lysates, which display substantial autofluorescence in the blue and green part of the spectrum.

## Results and Discussion

For the MST experiments in this study, the MST optimized dyes NT647 and NT547 were conjugated to tris-NTA. For the blue channel, the dye Oregon Green® 488^25^ was chosen. BLUE (OregonGreen® 488) is a derivate of fluorescein, the dyes RED (NT647) and GREEN (NT547) have distinct structures. The synthesis of DYE-tris-NTA constructs and the loading of the dyes with Ni(II) ions was performed according to previously described protocols^24,25^. Concentrations of DYE-tris-NTA conjugates were determined photometrically and the dyes were stored at −20 °C until further use.

### The affinity of DYE-tris-NTA for oligohistidine sequences

**Figure 2:**
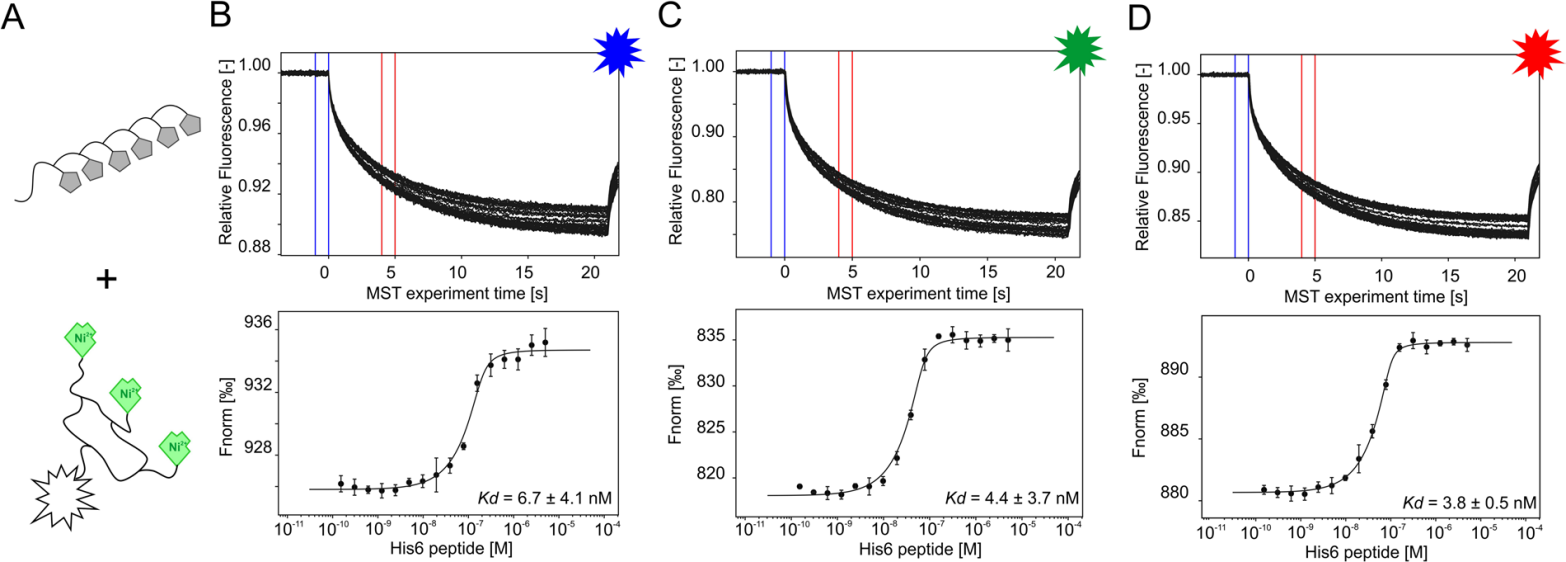
MST interaction analysis of His_6_-peptide against different tris-NTA fluorophores. (**A**) Schematic representation of DYE-tris-NTA interaction with His_6_-peptide. (**B,C,D**) MST traces (top) and dose-response curves (bottom) of His_6_-peptide titrated against tris-NTA conjugated dyes. (**B**) BLUE-tris-NTA (**C**) GREEN-tris-NTA and (**D**) RED-tris-NTA. 25 nM of DYE-tris-NTA was added to a 16-step serial dilution of His_6_-peptide. Mean values of three independent experiments are shown. Error bars indicate the standard deviation. MST experiments were carried out at medium MST power at 25 °C. LED power was set to 20% (**B**), 100% (**C**) and 40% (**D**). The resulting dose-response curves were fitted to a one-site binding model to extract K_d_ values; the standard deviation was calculated using the K_d_ values from each independent experiment. Fnorm = normalized fluorescence.

**Figure 3:**
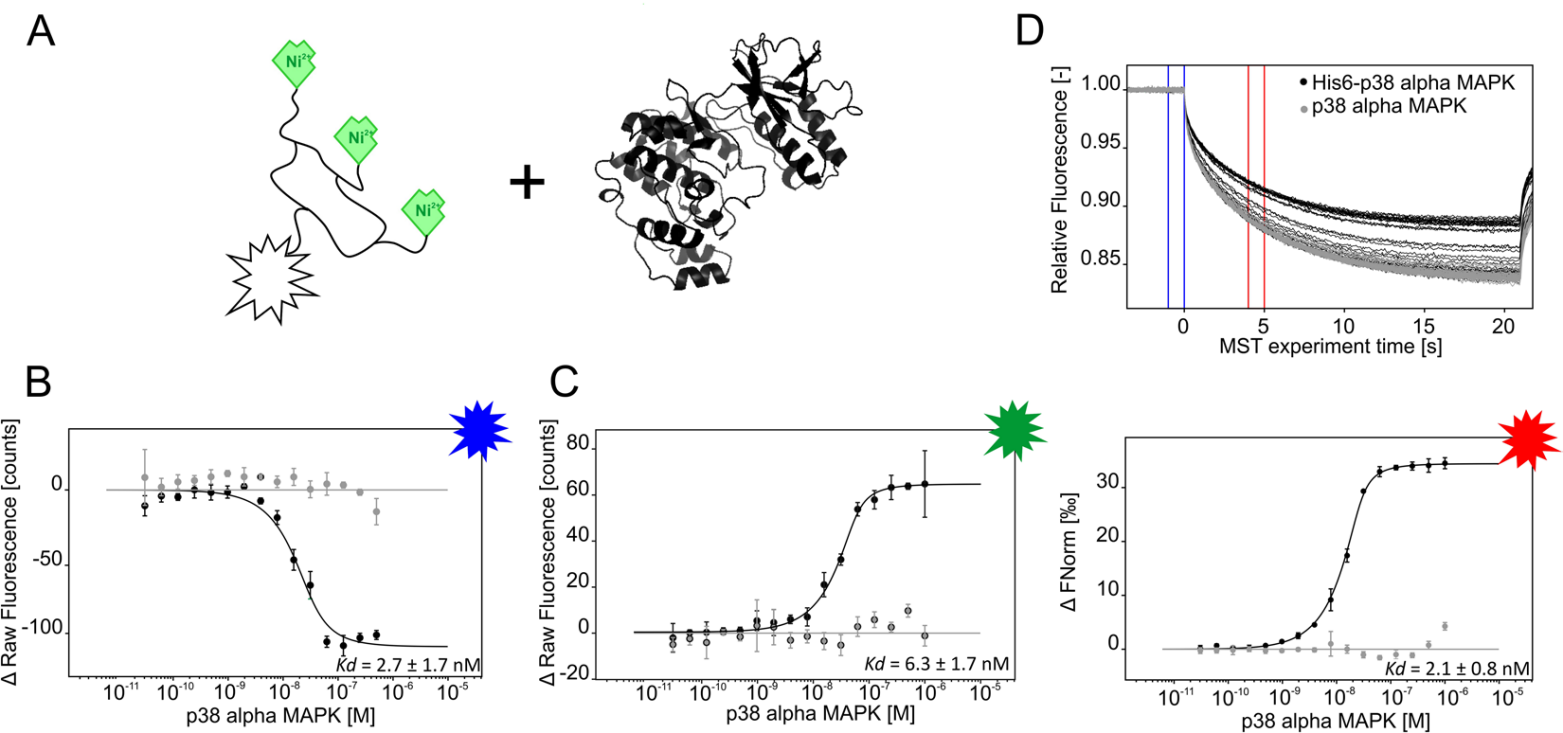
MST analysis of tris-NTA fluorophores interacting with p38α. (**A**) Schematic representation of tris-NTA-fluorophores and p38α (PDB: 1A9U). (**B,C**) Dose-response curves for BLUE-tris-NTA and GREEN-tris-NTA against His_6_-p38α (black) and p38α (grey) (n = 2). (**D**) MST traces (top) and dose-response curve (bottom) of His_6_-p38α (black) and p38α (grey) towards RED-tris-NTA (n = 3). All resulting dose-response curves were fitted to a one-site binding model to obtain K_d_ values. Error bars indicate the standard deviation. MST experiments were performed at a LED power of 60% (**A)**, 100% (**C**) and 40% (**D**) and at medium MST power. Fnorm = normalized fluorescence.

A high affinity of DYE-tris-NTA for oligohistidines is a prerequisite for labeling of proteins for the quantification of intermolecular interactions with MST. We therefore determined the affinity between the DYE-tris-NTA and two oligohistidine sequences: a His_6_-peptide and the MAP kinase p38 alpha (p38α) fused to an N-terminal His_6_-tag. As expected^24^, BLUE-, GREEN- and RED-tris-NTA displayed high affinity for the His_6_-peptide (6.7 ± 4.1 nM, 4.4 ± 3.7 nM and 3.8 ± 0.5 nM, respectively, Fig. 2). Comparable affinities were measured for the His_6_-tag fused to the N-terminus of p38α, yielding K_d_ values of 2.7 ± 1.7 nM for BLUE-tris-NTA, 6.3 ± 1.7 nM for GREEN-tris-NTA and 2.1 ± 0.8 nM for RED-tris-NTA, respectively (Fig. 3). These binding affinities are in excellent agreement with previously published K_d_ values of tris-NTA/His-tag interaction^24^. Slight differences in the K_d_ values between His_6_-peptide and His_6_-p38α can be explained with differences in the accessibility and the electrostatics due to structural characteristics of the protein and the fluorophores.

Notably, the binding of BLUE-tris-NTA and GREEN-tris-NTA to His_6_-p38α resulted in a ligand-induced fluorescence change. To exclude the possibility of nonspecific interactions between these dyes and the ligand, we performed an EDTA/His_6_-peptide (ECP) test, which quantifies ligand-induced fluorescence changes while using DYE-tris-NTA labeling. It consists of two subtests that must be performed to unambiguously distinguish between fluorescence changes caused by interaction and those caused by non-specific effects. In the case of His-tag labeling non-specific effects can be caused by interaction of a ligand with the His-tag bound tris-NTA dye (His_6_-peptide test) or by ligand-induced aggregation or adsorption to labware (EDTA test). Nonspecific interaction between BLUE-tris-NTA and GREEN-tris-NTA and the ligand was excluded based on this test (Supplementary Figure S1). Hence, the fluorescence signal was used for evaluation of binding data (Fig. 3B and C). In addition, for the p38α protein lacking His_6_-tag no binding was detected with any DYE-tris-NTA (Fig. 3).

### Stability of DYE-tris-NTA binding to His-tags

The Ni(II)/His-tag interaction is reversible upon addition of competitor substances such as histidine or imidazole. This feature is useful in some contexts, since it enables removal of immobilized molecules from microarray surfaces or elution of purified proteins from column-chromatography^32,33^. However, in the context of protein labeling for binding studies, disruption of the Ni(II)/His-tag interaction should be avoided, since it results in dissociation of the dye. To investigate potentially interfering buffer components, we systematically screened a set of common additives with respect to their effects on tris-NTA labeling. To this end, His_6_-peptide was titrated against RED-tris-NTA, while varying the concentration of different additives in the assay buffer PBST. These additives are listed in Supplementary Table S1, together with the highest concentration tested and their maximum tolerable concentration for the tris-NTA labeling approach. The maximum allowed assay concentration was defined as the highest concentration that did not alter the K_d_ value, whereas a slight decrease in the signal-to-noise ratio or in the binding amplitude was tolerated. In general, chelating agents such as ethylenediaminetetraacetic acid (EDTA) and ethylenglycol-bis(aminoethylether) (EGTA) or the ionic detergent sodium dodecyl sulfate (SDS) should be avoided. In addition, Tris-based assay buffers are known to exhibit some ion-complexing capacities^34,35^, therefore a caution and additional pretests are recommended when used for DYE-tris-NTA labeling. The use of divalent metal ions as Zn(II), Co(II) and Ni(II) as additives is not recommended, because they interfere with the labeling. Bovine serum albumin (BSA) and various proteins without His-tag showed no interference at any of the tested concentrations. In general, the tris-NTA labeling method turned out to be highly robust toward buffer additives, even regarding competitor substances such as imidazole or histidine. Here, only concentrations higher than 1 mM showed interference with the labeling reaction. Additionally, reducing agents such as tris(2-carboxyethyl)phosphine hydrochloride (TCEP) and dithiothreitol (DDT) can be used in the labeling buffer as well. This insensitivity and robustness of DYE-tris-NTA thus mostly allows labeling of proteins directly in their storage buffers, without the need for buffer exchange.

### Determination of ligand binding affinity using DYE-tris-NTA labeled target proteins

**Figure 4:**
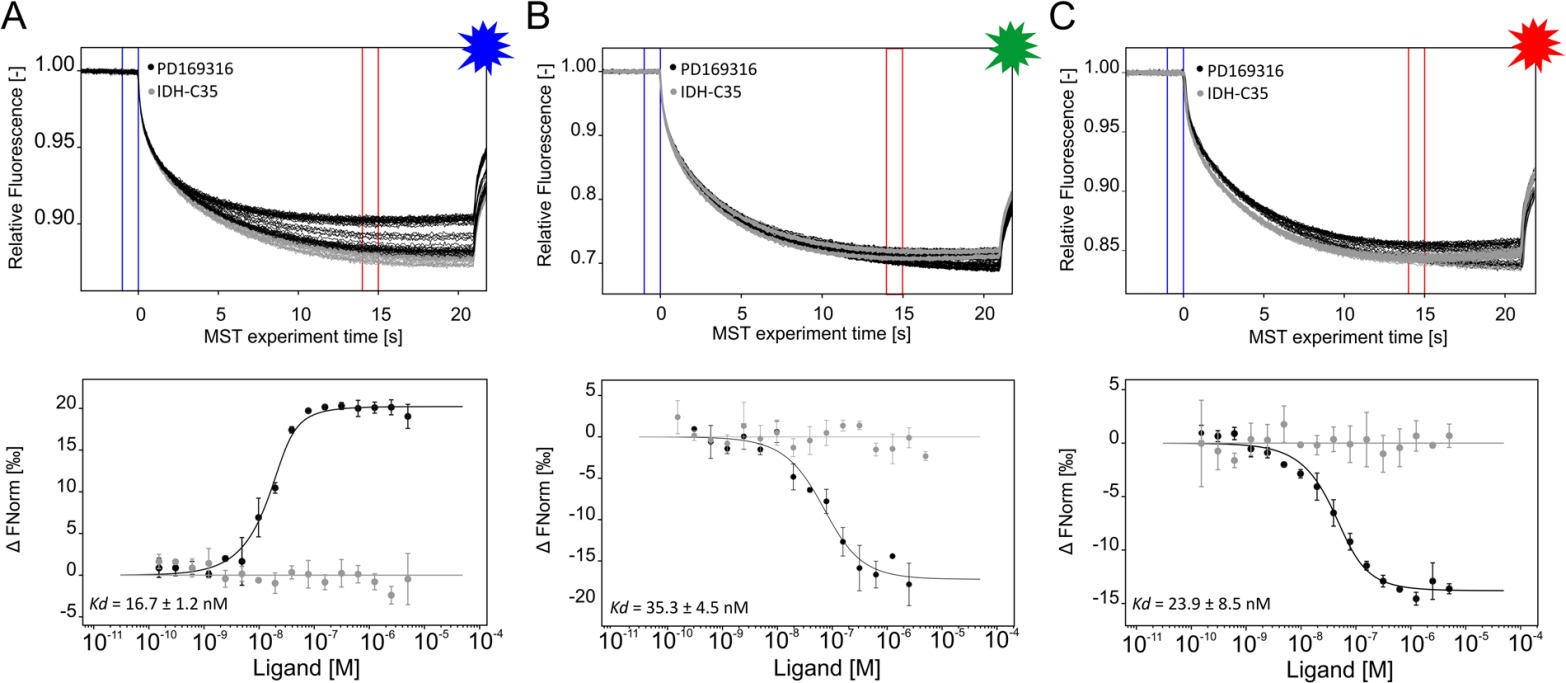
The interaction of p38α protein with small molecule inhibitors. MST traces (top) and dose-response-curves (bottom) for labeled His_6_-p38α (BLUE-tris-NTA (**B**), GREEN-tris-NTA (**C**) and RED-tris-NTA (**A**)) against PD169316 (black) or IDH-C35 (grey). The resulting dose-response curves were fitted to a one-site binding model for K_d_ determination. All measurements were done in triplicates, error bars indicate the standard deviation. MST experiments were performed at high MST power and LED power of 60%,100% and 20% (from left to right). Fnorm = normalized fluorescence.

A high affinity of DYE-tris-NTA for His-tags is a prerequisite for efficient stoichiometric labeling of His-tagged proteins for MST measurements. Because of all our DYE-tris-NTA candidates showed an affinity in the low nM range, we proceeded with the determination of ligand binding affinity using DYE-tris-NTA labeled target proteins. For a protein-small molecule interaction, His_6_-p38α was labeled with all three DYE-tris-NTA candidates separately. To ensure that virtually all dye is bound to the protein, we labeled the protein at a ratio of 1:2 (dye:protein) and incubated the protein/dye mixture for 30 min at room temperature to enable complete binding of the DYE-tris-NTA to the protein. As evident from the experiment depicted in Fig. 3, when we use 25 nm of DYE-tris-NTA, the binding of the DYE-tris-NTA reaches the saturation at the concentration of p38α of about 50 nM. With further increasing of the protein concentration, no additional increase in the binding of the dye is observed. This means that all dye is bound to the protein at a ratio of about 1:2. Based on this finding, efficient labeling was achieved and no separation of unbound dye was required. We proceeded immediately to the next step and added the labeled target protein to a dilution series of PD169316, a known selective inhibitor of p38α^36,37^. The ligand AGI-5198 (IDH-C35), a potent inhibitor of isocitrate dehydrogenase 1, served as a negative control. For PD169316 we measured K_d_ values of 16.7 ± 1.2 nM, 35 ± 5 nM and 24 ± 9 nM for BLUE-, GREEN- and RED tris-NTA, respectively, which is in accordance with published values^38^ (Fig. 4).

**Figure 5:**
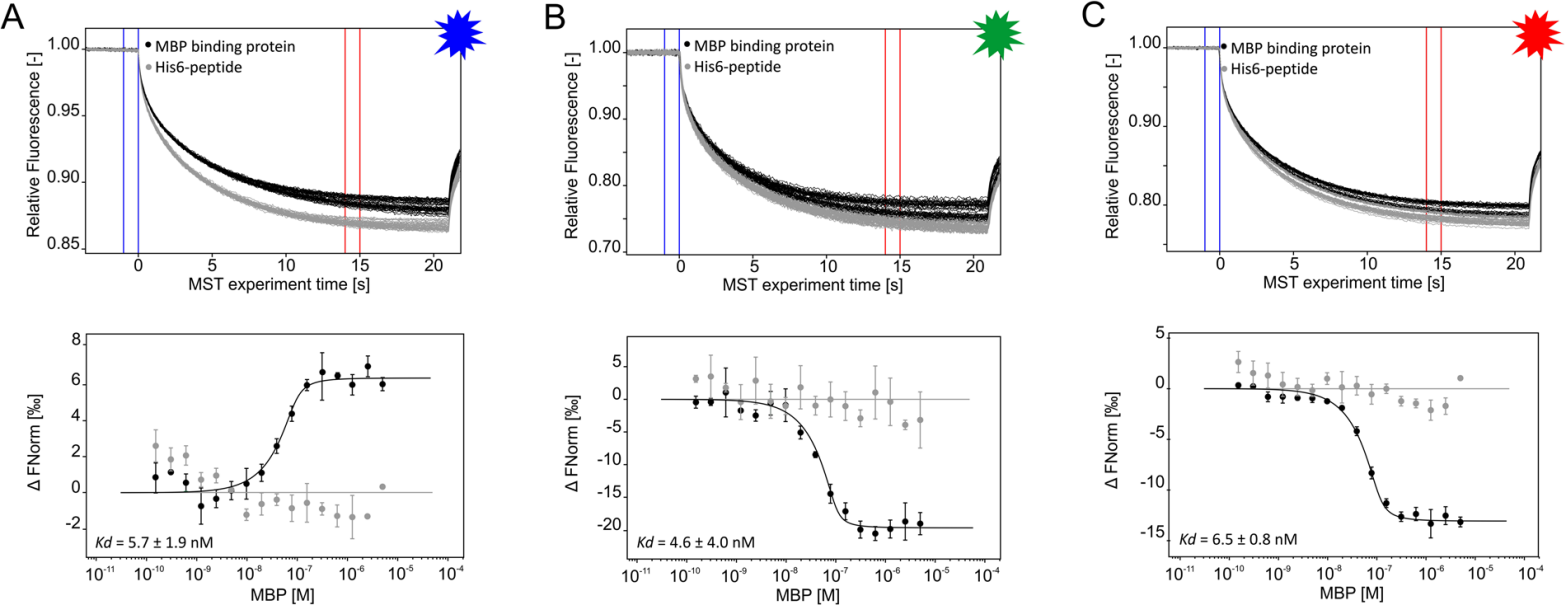
MST traces (top) and dose-response curves (bottom) of MBP towards BLUE-tris-NTA-MBP-binding protein (**A**), GREEN-tris-NTA-MBP-binding protein (**B**) and RED-tris-NTA-MBP-binding protein (**C**). The resulting dose-response curves were fitted to a one-site binding model to extract K_d_ values. All experiments were done in triplicates. Error bars indicate the standard deviation. MST experiments were performed at a LED power of 60% (**A**), 100% (**B**) and 40% (**C**) and at high MST power. Fnorm = normalized fluorescence.

As a second example, the interaction between maltose-binding-protein (MBP) and MBP-binding protein was analyzed. This 15 kD V_H_H binds MBP of *E. coli* with high affinity (ChromoTek GmbH, unpublished data). For MST affinity analysis, His_6_-tagged MBP-binding protein was labeled with all three DYE-tris-NTA conjugates separately and added to a serial dilution of MBP in PBST buffer. The K_d_ values measured were 6 ± 2 nM for BLUE-tris-NTA, 5 ± 4 nM for GREEN-tris-NTA and 7 ± 1 nM for RED-tris-NTA (Fig. 5). No binding was detected for labeled His_6_-peptide against titrated MBP, which underscores the high specificity of the MBP-binding protein for its ligand MBP. Among all DYE-tris-NTA candidates, RED-tris-NTA showed the best signal-to-noise ratio.

As is evident from Figs 3 to 5, differences in MST binding curve direction can be observed between the three DYE-tris-NTA candidates. For RED-tris-NTA and GREEN-tris-NTA, the unbound state of the protein exhibits the smallest changes in fluorescence; while for BLUE-tris-NTA, the bound state of the protein exhibits the smallest changes in the fluorescence. As mentioned previously, RED- and GREEN-tris-NTA are highly similar regarding their chemical structures, whereas BLUE-tris-NTA belongs to a different family of dyes. These molecular properties likely result in different MST signals of labeled proteins in the unbound and bound state and thus reverse the direction of the sigmoidal binding curve.

### Determination of ligand binding affinity by labeling of His_6_-tagged target proteins in crude cell lysate

The determination of ligand binding affinity directly in complex sample matrices like crude cell lysate, cell-free expression systems and blood serum offers several advantages. This approach is not only faster and more cost-effective, but it also enables studies with proteins in their natural environment. As the degree of autofluorescence from cell lysate components is higher for green and blue spectral regions, we decided to use RED-tris-NTA for all ligand binding studies in cell lysates.

As a first example, p38α-mNeonGreen-His_6_ and mNeonGreen-His_6_ were both expressed in mammalian cells for direct labeling in cell lysate using RED-tris-NTA. Labeling products were then used to quantify the interaction between p38α and SB203580. Thereby, mNeonGreen-His_6_ served as a negative control to verify the high specific binding of the small inhibitory compound to p38α. Further, the same interaction was quantified using mNeonGreen-His_6_-p38α as a target. Fluorescent proteins like mNeonGreen can be used as a fluorescent label for MST affinity analysis, which is performed directly in the cell lysate.

**Figure 6:**
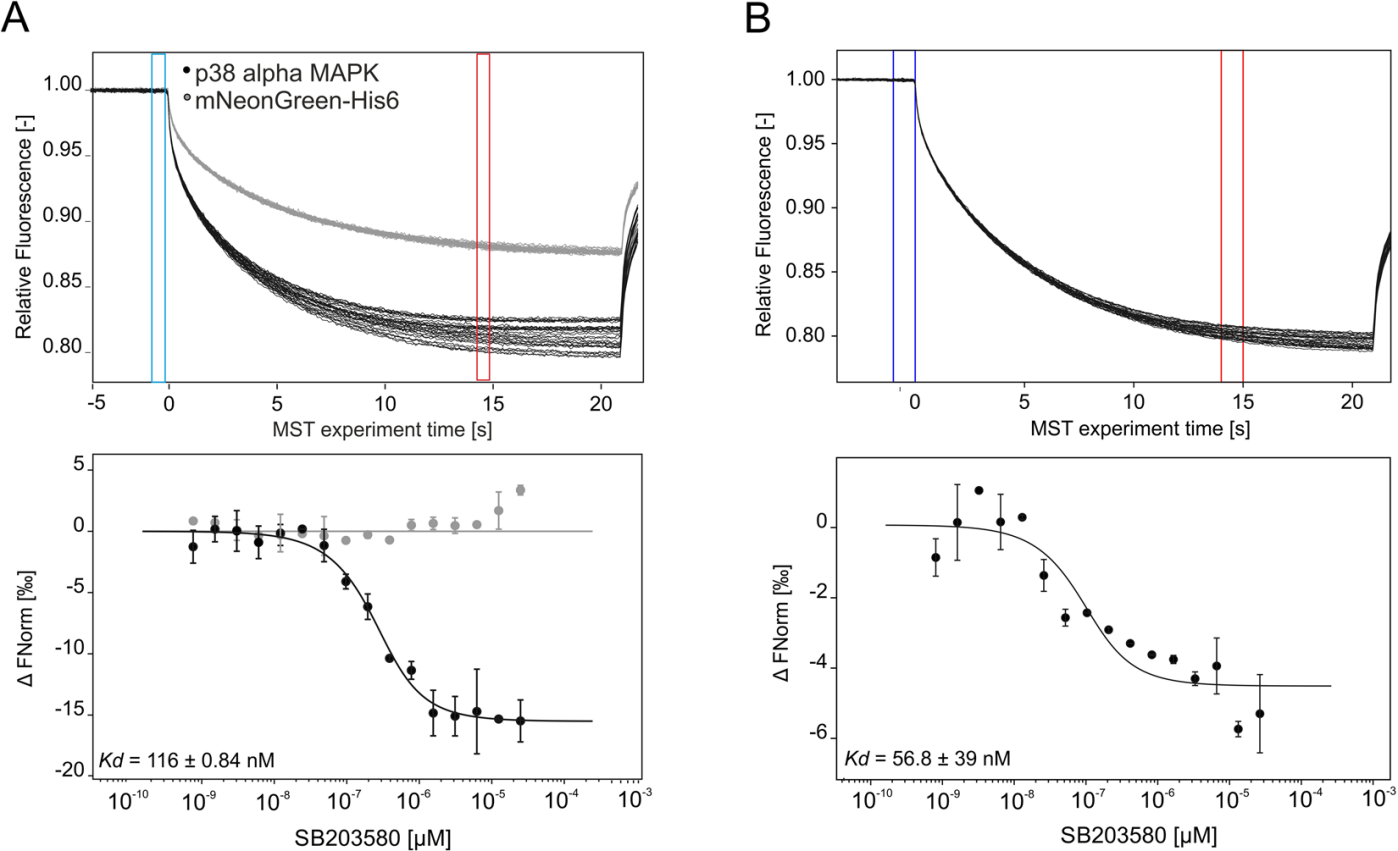
MST traces (top) and dose-response curve (bottom) of the interaction analysis of p38α against SB203580 in HeLa cell lysate in two different detection channels. (**A**) His_6_-p38α was labeled in HeLa cell lysate to determine its binding affinity toward SB203580 (black, n = 3). MST experiments were carried out at 100% LED and high MST power. mNeonGreen-His_6_ served as negative control and did not yield a binding curve (grey, n = 2). Here, 20% LED and high MST power were used. B) p38α-mNeonGreen-His_6_ against SB203580 (n = 2). Experiments were carried out at 20% LED and high MST power. The extracted K_d_ value was 56.8 ± 39 nM. Fnorm = normalized fluorescence.

To first determine the optimal dye-to-protein ratio for this labeling approach, first the concentration of p38α in cell lysate was experimentally determined. Therefore, cell lysate was titrated against a constant concentration of RED-tris-NTA and MST was carried out. Knowing the K_d_ value of this interaction from the experiments described above (Fig. 3), the concentration of p38α in the cell lysate could be determined as described in the Method section. Assuming the expression level of mNeonGreen-His_6_ to be like that of mNeonGreen-His_6_-p38α, the same amount of fluorophore was added to cell lysate containing mNeonGreen-His_6_, which served as negative control. For the MST experiment, a serial dilution of SB203580 was prepared using PBST buffer and a constant amount of labeled p38α-mNeonGreen-His_6_ or labeled mNeonGreen-His_6_ was added to all dilution steps. Only cell lysate containing labeled p38α showed clear binding towards SB203580 with a K_d_ of 116 ± 0.84 nM, while no binding could be detected for the p38α-free measurement (Fig. 6A). When mNeonGreen-His_6_-p38α was used as the target, much higher noise level was observed at simultaneously smaller binding amplitude (Fig. 6B), which consequently resulted in a less reliable fit of the K_d_ value. Nevertheless, the estimated K_d_ of 56.8 ± 39 nM is comparable to the K_d_ determined with RED-tris-NTA labeled His_6_-p38α.

**Figure 7:**
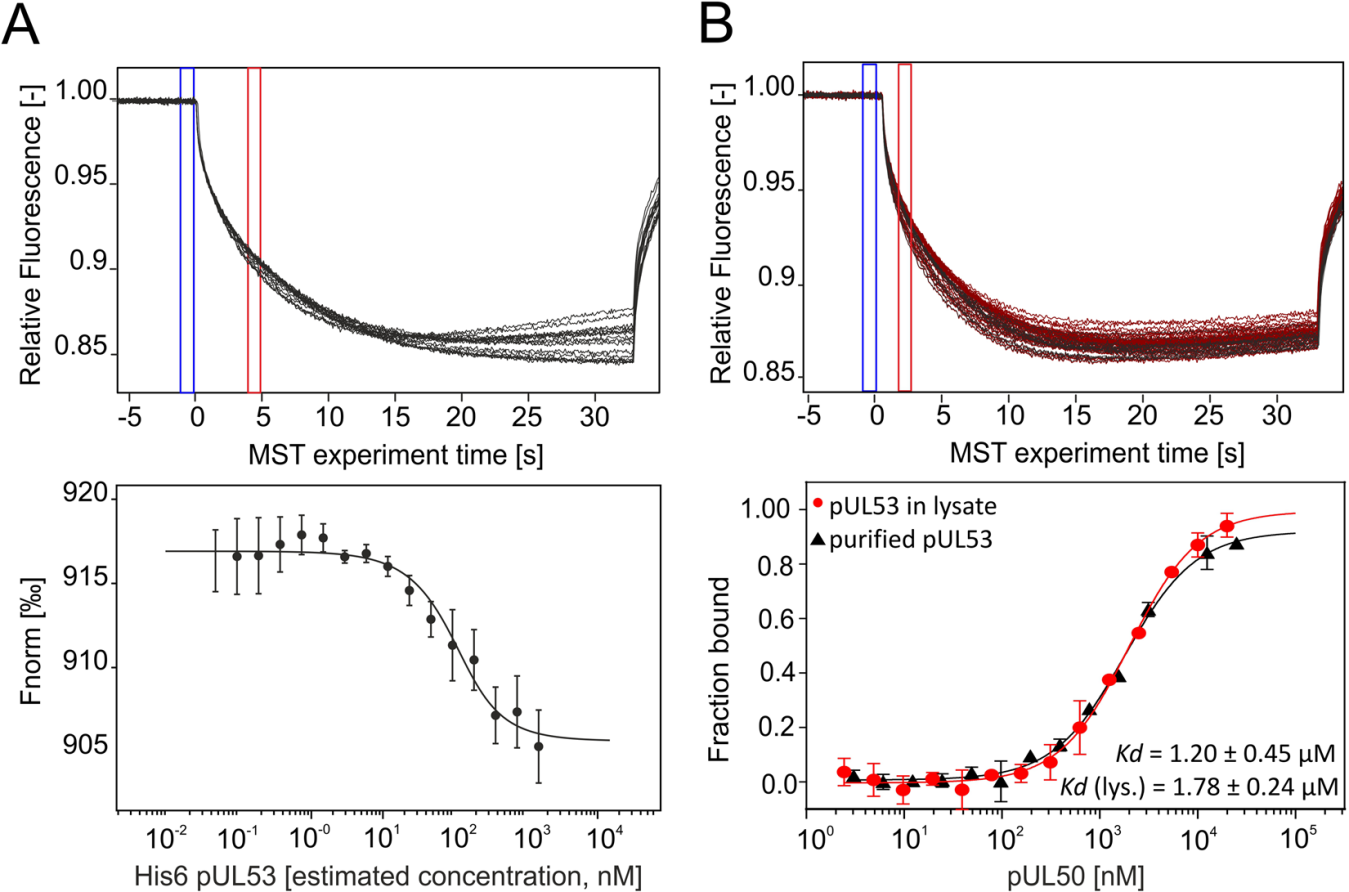
MST affinity analysis of RED-tris-NTA toward His_6_-pUL53 containing cell lysate and of labeled pUL53 toward pUL50 in *E. coli* lysate. MST traces (top) and dose-response curves (bottom) for His_6_-pUL53 against RED-tris-NTA (**A**) and RED-tris-NTA labeled pUL53 against pUL50 (B) (n = 3). (**A**) The interaction between RED-tris-NTA and His_6_-pUL53 was measured directly in *E. coli* lysate at LED 40% and high MST power. (**B**) MST traces of His_6_-pUL53 RED-tris-NTA in *E. coli* lysate (top) and comparison of the binding affinity of pUL50 toward labeled His_6_-pUL53 measured either with purified His_6_-pUL53 (black) or with His_6_-pUL53 in crude lysate (red) (bottom). Measurements were performed at 40% LED and medium MST power. Fnorm = normalized fluorescence.

In the next experimental settings, we compared the MST measurements, in which either the purified protein or the protein in crude cell lysate was employed. Therefore, we used two proteins His_6_-pUL53 and pUL50, which form the core nuclear egress complex of human cytomegalovirus (HCMV)^39^. First we tested the binding affinity of RED-tris-NTA towards His_6_-pUL53 in *E. coli* cell lysate. For this purpose, protein-containing cell lysate was titrated and a constant amount of RED-tris-NTA was added to all dilution steps. Figure 7A shows the MST data for this interaction. The binding curve was further used to roughly estimate the concentration of His_6_-pUL53 in the cell lysate to further determine the optimal dye concentration for protein labeling. To quantify the interaction between RED-tris-NTA labeled His_6_-pUL53 and pUL50, a serial dilution of the ligand was prepared using PBST buffer and a constant amount of labeled target protein was added to all dilution steps. As a control, pUL53 was purified after expression in *E. coli*, labeled using RED-tris-NTA and added to a serial dilution of pUL50 in HEPES buffer. K_d_ values of 1.2 ± 0.5 μM for the purified protein and 1.8 ± 0.2 μM for the measurements in crude cell lysate were obtained. These two values are in good agreement with each other and differ only slightly from the K_d_ value reported by Sam *et al*. using ITC measurements (K_d_ = 0.29 µM)^40^. The likely reason for this is that formation of the heterodimeric His_6_-pUL53:pUL50 complex is preceded by the dissociation of homodimeric His_6_-pUL53 into monomers. Hence, the K_d_ values measured here represent apparent affinities, and are thus concentration-dependent.

These two examples highlight the versatility, specificity and robustness of the RED-tris-NTA/His-tag system for the use in complex sample matrices such as cell lysate. Measured K_*d*_ values were in good agreement with published values and undistinguishable from the binding affinities determined in MST experiments using purified proteins.

## Conclusions

MicroScale Thermophoresis (MST) is routinely used for the quantification of molecular binding events and can even be used with complex biological matrices like cell lysate and serum. With the intention to develop a near-native labeling strategy for MST measurements, we exploited the tris-NTA/His-tag system, which comprises one of the smallest high-affinity recognition elements known to date. The conjugation of BLUE (OregonGreen® 488), GREEN (NT547) and RED (NT647) to tris-NTA resulted in fluorescence probes with high affinity and selectivity for oligohistidine tags, an ideal tool for site-selective labeling of proteins for quantitative characterization of intermolecular interactions by MST. All investigated DYE-tris-NTA conjugates displayed a high affinity for oligohistidine-tags. The RED-tris-NTA conjugate was identified as the optimal dye conjugate, requiring only low LED power and yielding the best signal-to-noise ratios. This conjugate was also successfully used for the labeling of oligohistidine-tagged proteins in crude cell lysate, which allowed the determination of binding affinity for a binding partner directly in lysate. As outlined in this study, the compatibility of RED-tris-NTA with complex sample matrices such as cell lysate has two major benefits: firstly, it permits the study of biomolecular interactions in near-native environment, allowing a more physiologically realistic assessment. And secondly, it may eliminate the need for protein purification for many MST assay setups, enabling shorter workflows and easier investigation of difficult-to-purify proteins. Combined with its high specificity for His-tags, this labeling strategy offers numerous advantages for protein labeling.

## Materials and Methods

### Materials

Chemicals for the synthesis of tris-NTA conjugated fluorophores and small molecule inhibitors were purchased from Sigma-Aldrich: dimethylformamide (DMF; Cat. No. 227056), ethyldiisopropylamine (EDIPA; Cat. No. 03440), nickel(II) chloride (NiCl_2_; Cat No. 339350), PD169316 (Cat. No. P9248), AGI-5198 (IDH-C35; Cat. No. SML0839) and SB203590 (Cat. No. S8307). Fluorophores (NHS ester) were obtained from NanoTemper Technologies GmbH (NT647; Cat. No. MO-L001 and NT547; MO-L002) or from ThermoFisher Scientific (Oregon Green® 488; Cat. No. O6149). Proteins were obtained from CRELUX GmbH (p38α), from ChromoTek GmbH (MBP-binding protein; Cat. No. mbt-250) or from antibodies-online GmbH (MBP; Cat. No. MBP0801). His_6_-peptide was purchased from APExBIO (Cat. No. A6006).

All measurements were carried out on a Monolith NT.115 device (NanoTemper Technologies GmbH), equipped with a RED/GREEN or BLUE detection channel.

### Synthesis and preparation of tris-NTA conjugated fluorophores

OregonGreen® 488-tris-NTA was prepared as previously published^25^. NT647 and NT547-tris-NTA conjugates were synthesized and characterized as following: tris-NTA modified by an aminocaproic acid (tris-NTA, MW: 1048 g/mol) was synthesized as previously described^24^ to yield tris-NTA-ACA. 5.9 mg tris-NTA-ACA was dissolved in 100 µl dry DMF, followed by addition of 12 µl EDIPA. 3.0 mg NHS ester of fluorophores was separately dissolved in 100 µl dry DMF. Solutions were mixed and stirred overnight (20 h) at room temperature under the protection of N_2_ atmosphere in darkness. After addition of 100 µl H_2_O, the reaction mixture was continuously stirred for 1 h to quench unreacted NHS groups. The mixture was diluted in 20 ml of 0.1% TFA/water and was loaded on a C_18_ reverse phase HPLC column (Vydac 218TP, 250 × 4.6 mm) for purification using a 0–70% acetonitrile gradient in 0.1% TFA/water (1/6 of the reaction per run). The DYE-tris-NTA conjugates were eluted at ~45% acetonitrile. The purified DYE-tris-NTA fractions of each dye were pooled together and lyophilized as blueish, orange or pink powder and stored at −20 °C. Obtained products were verified by the MS-ESI analyses.

For the nickel loading, the obtained DYE-tris-NTA- were dissolved in 20–50 mL of 10 mM HEPES buffer pH 7.5 to a final concentration of 0.1 mM or less. NiCl_2_, in a final concentration of 5 mM was added to this solution for loading Ni(II) ions on the NTA groups. After 15 min, the solution was loaded onto a 1 ml anion-exchange column (Hitrap Q, GE Healthcare) and eluted with a gradient of 0–600 mM sodium chloride in 10 mM HEPES buffer, pH 7.5. The Ni(II)-loaded tris-NTA-fluorophores were eluted at ~300 mM sodium chloride. For each dye, the fractions from the elution peak were combined, and the concentrations were determined photometrically at 647 nm and 547 nm using an extinction coefficient of 250000 M^−1^cm^−1^ and 150000 M^−1^cm^−1^, respectively. The final products were aliquoted in black Eppendorf tubes and stored at −20 °C.

### Determination of DYE-tris-NTA binding affinity for oligohistidine tags

His_6_-peptide or His_6_-p38α (expression construct CJA3) were diluted in PBST buffer to a final concentration of 10 µM and 2 µM, respectively. This solution was used for a 16-step serial dilution in PBST buffer (137 mM NaCl, 2.5 mM KCl, 10 mM Na_2_HPO_4_, 2 mM KH_2_PO_4_, pH 7.4, 0.05% Tween-20) with 10 µl volume in each sample. Next, 10 µl of 50 nM dye, dissolved in PBST buffer, was added to all vials of the serial dilution. Samples were mixed by pipetting up and down and reaction was incubated for 30 min at room temperature in the dark, before samples were loaded into Monolith NT.115 Capillaries. Samples were then transferred into the Monolith NT.115 device and MST experiments were carried out at 40% (RED)/20% (BLUE)/100% (GREEN) LED and medium MST power for the His_6_-peptide measurements and at 40% (RED)/60% (BLUE)/100% (GREEN) LED and medium MST power for the His_6_-p38α studies.

### Labeling and MST measurements of purified His_6_-tagged proteins

Proteins (His_6_-p38α and His_6_-MBP-binding protein were diluted to 200 nM in PBST buffer (137 mM NaCl, 2.5 mM KCl, 10 mM Na_2_HPO_4_, 2 mM KH_2_PO_4_, pH 7.4, 0.05% Tween-20). Tris-NTA dyes were diluted in PBST buffer to a final concentration of 100 nM. 100 µl of protein was then mixed with 100 µl of each dye separately and the reaction mixtures were incubated for 30 min at room temperature in the dark.

Ligand dilution series: Small molecule inhibitors were stored in 100% DMSO at −20 °C. For the dilution series, a 10 µM solution of PD169316 or of AGI-5198 (IDH-C35) was prepared using PBST buffer (2% final DMSO concentration). This stock solution was used for the preparation of a 16-step serial dilution in PBST buffer, supplemented with 2% DMSO, with a final volume of 10 µl in each vial of the dilution series. For the protein-protein interaction, 5 µM of MBP diluted in PBST buffer was used as the highest ligand concentration of the 16-step dilution series, with a final volume of 10 µl in each reaction tube.

Then 10 µl of 100 nM RED/GREEN or BLUE labeled protein (p38α or MBP-binding protein) was added to all 16 vials and samples were mixed by pipetting up and down. Reactions were incubated for 30 min at room temperature away from light and then loaded into Monolith NT.115 Capillaries. Using the Monolith NT.115 device, MST was carried out at 20% (RED)/60% (BLUE)/100% (GREEN) LED and high MST power for p38α, and at 40% (RED)/60% (BLUE)/100% (GREEN) LED and high MST power for MBP-binding protein.

### Labeling and MST measurements of oligohistidine-tagged proteins in crude cell lysate

#### p38α against SB203580

The p38α protein sequence was obtained through reverse transcription on mRNA from A549 cells. Using In-Fusion Cloning technology (Takara Bio USA, Inc.) this p38α coding sequence was cloned in a pcDNA3.1 mammalian expression vector behind a CMV promotor and separated from the mNeongreen-His_6_-tag by the linker sequence ESGSGS. A pcDNA3.1 vector coding for only mNeongreen-His_6_ was used as a control. These two plasmids expressing mNeongreen-His_6_ with and without the p38α sequence were transfected into 3*10^6 HeLa cells using separate T-75 flasks. Cells were grown for 24 h reaching approximately 10*10^6 cells. Cells were pelleted by centrifugation and resuspended in 1 ml PBST buffer (137 mM NaCl, 2.5 mM KCl, 10 mM Na_2_HPO_4_, 2 mM KH_2_PO_4_, pH 7.4, 0.05% Tween-20), supplemented with protease inhibitors. At this step, the cells were disrupted using a Dounce homogenizer and centrifuged again at 14 000 × g for 30 min at 4 °C to remove cell debris. Obtained supernatant was diluted 1:10 in PBST, supplemented with protease inhibitors.

Concentration of His_6_-tagged protein in cell lysate was determined by the MST experiment as described in the Supplemental information, Fig. S2. The labeling of p38α-mNeonGreen-His_6_ and mNeonGreen-His_6_ in HeLa cells was carried out by mixing 100 µl of about 100 nM p38α-mNeonGreen-His_6_ or mNeonGreen-His_6_ with 100 µl of 100 nM NT647-tris-NTA dye in PBST buffer. Reaction mixture was incubated for 30 min at room temperature.

For the MST binding experiment, the stock solution of SB203580 (stored at 2.65 mM in 100% DMSO at −20 °C) was diluted 1:50 in PBST, reaching a concentration of 53 µM with 2% DMSO. This solution was used for a 1:1 serial dilution using 16 dilution steps and a final volume of 10 µl for each point of the dilution series. Afterwards 10 µl cell lysate was added to all steps of the dilution series, giving a final ligand concentration of 26.5 µM with 1% DMSO. Reaction was incubated for 30 min at room temperature, centrifuged at 14 000 g for 10 min at 4 °C and loaded into Monolith NT.115 MST Premium Capillaries. MST experiment was carried out using 100% or 20% LED power for the p38α containing sample and for the negative control, respectively, and high MST power for the NT.115 RED instrument. For the NT.115 blue device 20% LED and high MST power was used.

#### pUL53 against pUL50

For protein expression, a plasmid encoding a His_6_-tagged protein variant of pUL53 (residues 50 to 292 of human cytomegalovirus ORF-UL53 1-376)^39^ was transformed into BL21(DE3) cells and grown in LB medium in the presence of 100 mg/ml ampicillin and 32 mg/ml kanamycin at 33 °C until OD_600_ of 0.4. When the required OD was reached, 0.25 mM isopropyl -D-thiogalactopyranoside was added to induce protein expression. The culture was further incubated overnight at 20 °C. Cells were harvested by centrifugation, disrupted by sonication and resuspended in lysis buffer (50 mM phosphate buffer, pH 7.4, 300 mM NaCl) containing protease inhibitors, lysozyme, and DNase.

For affinity determination of His_6_-pUL35 against RED-tris-NTA, pUL53-containing cell lysate was diluted 1:10 in PBST buffer and a 16-step serial dilution was prepared. RED-tris-NTA was then added to all dilution steps with a final concentration of 25 nM. Samples were incubated for 30 min at room temperature in the dark, before they were loaded into Monolith NT.115 MST Premium Capillaries and loaded into the Monolith NT.115 device. MST experiment was carried out at 40% LED and high MST power.

Labeling of His_6_-pUL53 in *E. coli* lysate was carried out by diluting the lysate 1:10 in PBS-T buffer and adding RED-tris-NTA dye at a final concentration of 50 nM. The mixture was incubated for 30 min at room temperature. For the labeling of purified His_6_-pUL53, the protein was first purified from the *E. coli* cell lysate *via* a Ni-NTA affinity chromatography followed by a size exclusion chromatography step. After purification, His_6_-pUL53 was labeled by mixing100 µl of a 200 nM protein solution with 100 µl of 50 nM RED-tris-NTA using PBST as reaction buffer. Mixture was incubated for 30 min at room temperature in the dark.

For affinity analysis of purified and non-purified RED-tris-NTA His_6_-pUL53 against pUL50 (obtained as previously described)^39^, HEPES buffer (200 mM, 25 mM HEPES, 1 mM TCEP, pH 8.0) was used. The highest ligand concentration in the 16-step serial dilution series was 1 µM, with 10 µl volume in each titration step. 10 µl labeled protein was then added to all dilutions at a final concentration of 100 nM. Samples were mixed and loaded in Monolith NT.115 MST Premium Capillaries. MST experiments were carried out at 40% LED and 60% MST power.

#### Data acquisition and analysis

The data were acquired with MO.Control 1.5.3 (NanoTemper Technologies GmbH). Recorded data were analyzed with MO.Affinity Analysis 2.2.7 (NanoTemper Technologies GmbH). The MST on-time yielding the highest signal-to-noise ratio was used for the K_d_ determination. The data were fitted using a K_d_ fit model that describes a molecular interaction with a 1:1 stoichiometry according to the law of mass action. The *K*_*d*_ is estimated by fitting the Eq. 1:

1 $$f(c)=Unbound+(Bound-Unbound)\times \frac{c+{c}_{target}+{K}_{d}-\sqrt{{(c+{c}_{target}+{K}_{d})}^{2}-4c{c}_{target}}}{2{c}_{target}}$$

Where *f(c)* is the fraction bound at a given ligand concentration *c*; *Unbound* is the Fnorm signal of the target; *Bound* is the Fnorm signal of the complex; *K*_*d*_ is the dissociation constant or binding affinity; and the *c*_*target*_ is the final concentration of target in the assay.

### Data availability

The datasets generated during and/or analyzed during the current study are available from the corresponding author on reasonable request. The structural information on NT647 and NT547 is proprietary and cannot be disclosed.

## Electronic supplementary material

Supplementary Information
