# Supplementary material for: Near-native, site-specific and purification-free protein labeling for quantitative protein interaction analysis by MicroScale Thermophoresis
Source: Sci Rep. 2018 Mar 21;8:4977. doi: 10.1038/s41598-018-23154-3 (PMC5862892; doi:10.1038/s41598-018-23154-3)
Supplement: Supplementary file 1 — Supplementary Information [file 41598_2018_23154_MOESM1_ESM.pdf]

Supplementary Data

Near-native, site-specific and purification-free protein labeling for quantitative protein interaction analysis by MicroScale Thermophoresis

Tanja Bartoschik[1], Stefanie Galinec[1], Christian Kleusch[1], Katarzyna Walkiewicz[1], Dennis Breitsprecher[1], Sebastian Weigert[2], Yves A. Muller[2], Changjiang You[3], Jacob Piehler[3], Thomas Vercruysse[4], Dirk Daelemans[4] and Nuska Tschammer[1],*

[1] Dr. habil. Nuska Tschammer, Stefanie Galinec, Dr. Christian Kleusch, Dr. Dennis Breitsprecher, Dr. Katarzyna Walkiewicz, Tanja Bartoschik
NanoTemper Technologies GmbH
Floessergasse 4
81069 München, Germany

[2] Sebastian Weigert, Prof. Dr. Yves Muller
Division of Biotechnology, Department of Biology
Friedrich-Alexander University Erlangen/Nuremberg
Henkestr. 91, 91052 Erlangen, Germany

[3] Dr. Changjiang You, Prof. Dr. Jacob Piehler
Division of Biophysics, Department of Biology
University Osnabrück
Barbarastr. 11, 49076 Osnabrück, Germany

[4] Prof. Dr. Dirk Daelemans, Dr. Thomas Vercruysse
KU Leuven Department of Immunology and Microbiology, Laboratory of Virology and Chemotherapy
Rega Institute for Medical Research
Herestraat 49, 3000 Leuven, Belgium

*Correspondence to nuska.tschammer@nanotempertech.com

EDTA test

In MST experiments exhibiting ligand-dependent changes in initial fluorescence, the cause of this effect needs to be determined through specificity tests. In some cases, the interaction itself is causing the fluorescence changes, allowing data evaluation via initial fluorescence. In other cases, the effect is due to material loss such as adsorption of the fluorescent molecule to labware or protein aggregation.

The EDTA (ethylenediaminetetraacetic acid) test is used for assays with DYE-tris-NTA labeled samples and was performed according to instructions given in MO.Control software. For this, 7 µl of samples 1-3 and 14-16 were centrifuged for 10 min at 14 000 g and 4 °C, before 7 µl 50 mM EDTA (pH 7.4) solution was added to all six samples. Solutions were mixed by pipetting up and down and incubated for 30 min at 37 °C using a heating block. Afterwards, samples were loaded into Monolith NT.115 Capillaries and sample fluorescence was recorded at 25 °C and 60 % or 100 % LED power, for BLUE and GREEN respectively. Fluorescence intensities of duplicate measurements for the GREEN and the BLUE channel

are illustrated in figure S1. Here, the fluorescence intensities of samples 1-3 and 14-16 are presented before and after the addition of EDTA. As the high affinity of this interaction is dependent on the presence of Ni(II) ions complexed with the NTA molecule, the addition of a chelating agent like EDTA removes the Ni(II) ions from the tris-NTA dye, causing dissociation of the dye from the His-tagged protein. In case of a non-specific fluorescence decrease, the difference in initial fluorescence intensity will remain after addition of EDTA. In case of a binding specific fluorescence decrease, the initial fluorescence of all samples will be near-identical after EDTA addition, as seen in Figure S1.

In situations where interactions of DYE-tris-NTA labeled proteins with a third molecule are analyzed, the EDTA test is followed by the Control Peptide test. This is to detect fluorescence changes caused by the direct interaction of the third molecule with either the tris-NTA dye or the labeled target protein's His-tag. In this particular assay, only the interaction of the dye to the His-tagged protein was investigated, and the Control Peptide test was therefore not needed.

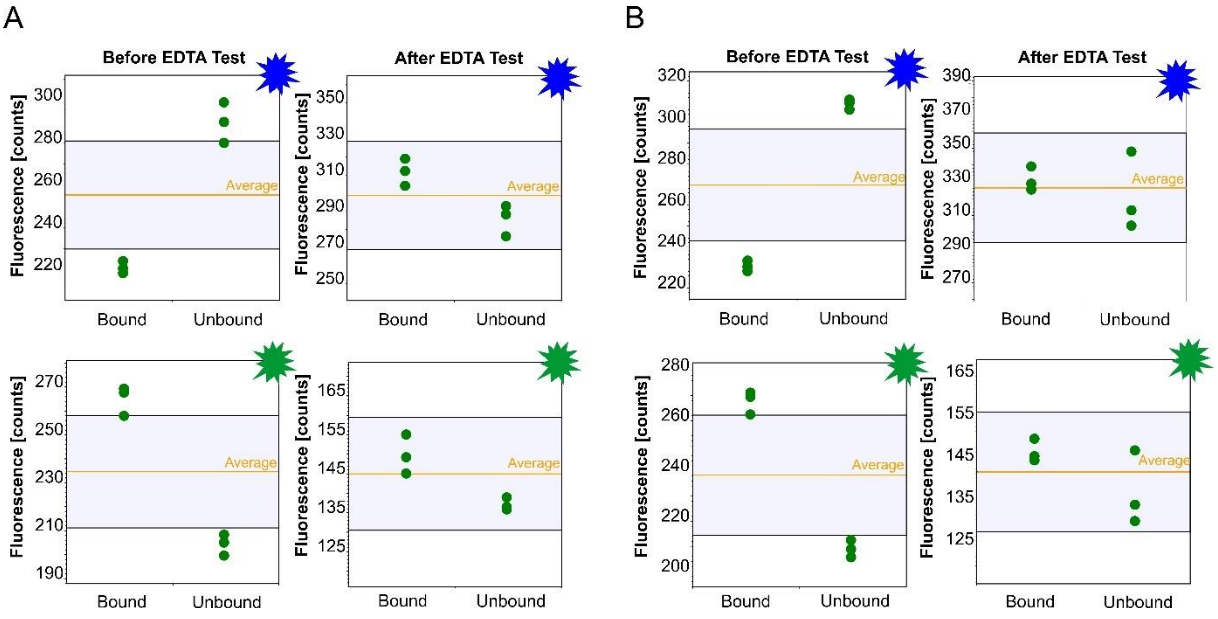

Figure S1: EDTA test to analyze the ligand-induced fluorescence changes observed for BLUE- and GREEN-tris-NTA binding to His_6-p38 alpha MAPK. Fluorescence intensities before and after addition of EDTA solution are illustrated for two both fluorophores, each measured in two biological replicates (A and B).

Table S1: Tested buffer additives to investigate robustness of tris-NTA labeling approach.

Buffer additive	Tested concentrations	Maximum allowed assay concentration
Histidine	0.25 mM/0.5 mM/1 mM	1 mM
Imidazole	0.25 mM/0.5 mM/1 mM	1 mM
EDTA, EGTA	0.25 mM/0.5 mM	0.5 mM
TCEP	0.25 mM/0.5 mM	0.5 mM*
DTT	0.25 mM/0.5 mM/1 mM/5 mM	5 mM
ß-mercapto-ethanol	0.25 mM/0.5 mM/1 mM	1 mM
GSH	0.25 mM/0.5 mM/1 mM/5 mM/10 mM	10 mM
GTP, GDP	0.25 mM/0.5 mM/1 mM	1 mM
AMP, ADP, ATP	0.25 mM/0.5 mM/1 mM/5 mM	5 mM
Glycerol	10%	10 %
Zn(II), Co(II), Cu(II)	100 nM	preloaded protein only**
Mg(II)	10 mM	5 mM***
Polyhistidine-tagged ligand		none

*NanoTemper Technologies GmbH recommends to avoid the use of TCEP with the red fluorophores in general.

**Zn^{2+}, Co^{2+}, Cu^{2+} ions compete for the binding with tris-NTA fluorophores. Thus only very low nanomolar concentrations are tolerated in the assay buffer. Additional pretests are required.

***Caution is required when using Mg(II) because the magnesium salts might be contaminated with significant amounts of divalent heavy metal ions (like Zn(II), Co(II), Cu(II)), which might interfere with the labeling.

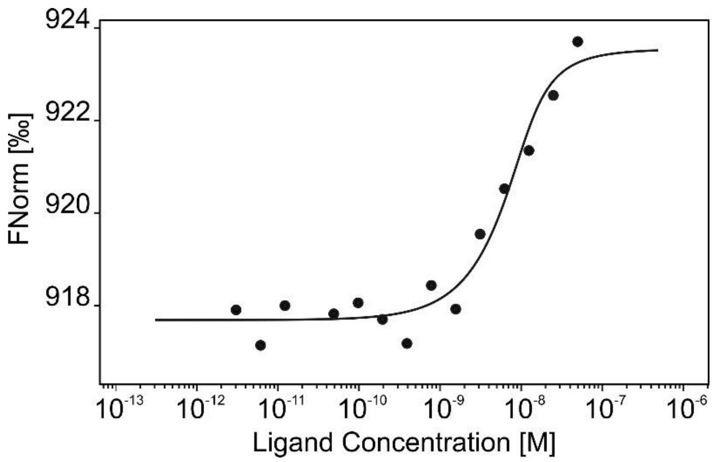

Figure S2: Dose-response curve of the interaction analysis of His$_6$-p38α against RED-tris-NTA in HeLa cell lysate, for the determination of protein concentration in the cell lysate. A serial dilution of His$_6$-p38α containing cell lysate was prepared and RED-tris-NTA was added at a constant concentration of 50 nM. Samples were then filled into Monolith NT.115 MST Premium Capillaries and MST experiment was carried out using 50 % LED and medium MST power.

Concentration of His$_6$-tagged p38α in cell lysate was determined as described. Briefly, a 1:1 serial dilution of p38α-mNeonGreen-His$_6$ containing HeLa cell lysate was prepared using non-transfected HeLa cell lysate as dilution buffer. 50 nM of RED-tris-NTA dye was added to all 16 dilution steps, followed by incubation for 30 min at room temperature. Samples were then filled into Monolith NT.115 MST Premium Capillaries and MST experiment was carried out using 50 % LED and medium MST power. MST data was evaluated after 10 s MST-on time. Resulting dose-response curve is illustrated in Figure S2. The data were fitted using a Kd fit model that describes a molecular interaction with a 1:1 stoichiometry according to the law of mass action. The K_d is estimated by fitting the equation 1:

$$f(c) = Unbound + (Bound - Unbound) \times \frac{c + c_{target} + K_d - \sqrt{(c + c_{target} + K_d)^2 - 4cc_{target}}}{2c_{target}} \quad [1]$$

Where *f(c)* is the fraction bound at a given ligand concentration *c*; *Unbound* is the Fnorm signal of the target; *Bound* is the Fnorm signal of the complex; K_d is the dissociation constant or binding affinity; and the c_{target} is the final concentration of target in the assay.

For the data set shown in the Figure S2, we set the K_d of RED-tris-NTA for the His-tagged p38alpha to 2.1 nM (as measuered for the purified protein) and the c_{target} on 25 nM. Taking into consideration the 1:1 dilution step, the concentration of the His-tagged p38alpha was estimated to be around 50 nM.

Table S2: Overview of experimental parameters.

Interaction	K_d with standard deviation	Amplitude*	S/N ratio**	Reduced χ^{2}***	Number of experiments
BLUE-tris-NTA against His_6-peptide	6.7 ± 4.1 nM	8.8	24.9	1.1	3
GREEN-tris-NTA against His_6-peptide	4.4 ± 3.7 nM	17.2	34.1	5.2	3
RED-tris-NTA against His_6-peptide	3.8 ± 0.5 nM	12.1	61.1	0.2	3
BLUE-tris-NTA against His_6-p38α	2.7 ±1.7 nM	100.3	18.6	3.1	3
GREEN-tris-NTA against His_6-p38α	6.3 ± 1.7 nM	63.6	34.4	0.3	3
RED-tris-NTA against His_6-p38α	2.1 ± 0.8 nM	34.4	91.0	1.4	3
His_6-p38α-BLUE-tris-NTA against PD169316	16.7 ± 1.2 nM	19.3	29.6	1.5	3
His_6-p38α-GREEN-tris-NTA against PD169316	35 ± 5 nM	17.1	13.5	1.5	3
His_6-p38α-RED-tris-NTA against PD169316	24 ± 9 nM	13.8	20.1	3.4	3
BLUE-tris-NTA-MBP-binding protein against MBP	6 ± 2 nM	6.3	12.7	14.9	3
GREEN-tris-NTA-MBP-binding protein against MBP	5 ± 4 nM	18.8	18.4	0.9	3
RED-tris-NTA-MBP-binding protein against MBP	7 ± 1 nM	13.0	42.9	3.8	3
His_6-p38α-RED-tris-NTA against SB203580 in HeLa cell lysate	116 ± 0.84 nM	16.1	22.4	3.0	3
mNeonGreen-His_6-p38α against SB203580 in HeLa cell lysate	56.8 ± 39 nM	4.6	6.3	26.4	2
RED-tris-NTA toward His_6-pUL53 containing cell lysate	not determined	11.3	16.2	0.4	4
RED-tris-NTA labeled pUL53 against pUL50 in *E.coli* lysate	1.8 ± 0.2 μM	10.1	42.3	1.5	3
RED-tris-NTA labeled pUL53 against pUL50 (purified)	1.2 ± 0.5 μM	12.2	26.6	3.8	2

*

$$Response\ Amplitude = |unbound - bound| \qquad [2]$$

Where unbound and bound are the respective estimated values from the fit. “Unbound” is the plateau at very low concentrations of ligand (also called baseline), while “bound” is the plateau at very high concentrations of ligand (also called saturation).

** The signal-to-noise is calculated by dividing the response amplitude by the noise [3]. The noise is calculated as the standard deviation of the residuals from the fit.

$$S/N = \frac{Response\ Amplitude}{\sqrt{\frac{\sum_i (r_i - \bar{r})^2}{n-1}}} \qquad [3]$$

Where r_i denotes the residual of the fit at a given data point and $\bar{r}$ the average of all residuals. The number of data points is given by n.

The signal to noise is a good parameter from which to judge data quality. A value of more than 5 is desirable while a value of more than 12 corresponds to an excellent assay.

*** This value is only calculated for merge sets that contain two or more replicates [4].

$$\chi^2 = \sum_i \frac{(m_i - y_i)^2}{\sigma_i} \qquad [4]$$

Where m_i denotes the y-values of the fitted curve, y_i denotes the averaged raw-data y-values and σ_i denotes the standard deviation of the averaged raw-data y-values.

The reduced χ^2 is then defined as [5]

$$\chi^2{}_{red} = \frac{\chi^2}{\nu} \qquad [5]$$

With the residual degree of freedom $\nu = n - m$; n is the number of data points and m is the number of parameters that are fitted (four parameters for both, Kd- and Hill-model, except any parameters are fixed).

In MO.AffinityAnalysis the reduced χ^2 can become quite large. The reason for this is that replicates are often very similar. This yields a small standard deviation. Since we divide by these small values, the number can become quite high. Therefore, the absolute value of the reduced χ^2 alone is not a useful parameter from which to judge data quality. It is however very useful for comparing data quality between replicates or comparable samples. In such cases, the smaller χ^2 for one particular dataset in comparison to other datasets, the better the data quality.
